# Flow starvation during square-flow assisted ventilation detected by supervised deep learning techniques

**DOI:** 10.1186/s13054-024-04845-y

**Published:** 2024-03-14

**Authors:** Candelaria de Haro, Verónica Santos-Pulpón, Irene Telías, Alba Xifra-Porxas, Carles Subirà, Montserrat Batlle, Rafael Fernández, Gastón Murias, Guillermo M. Albaiceta, Sol Fernández-Gonzalo, Marta Godoy-González, Gemma Gomà, Sara Nogales, Oriol Roca, Tai Pham, Josefina López-Aguilar, Rudys Magrans, Laurent Brochard, Lluís Blanch, Leonardo Sarlabous, the BEARDS study investigators, Laurent Brochard, Irene Telias, Felipe Damiani, Ricard Artigas, Cesar Santis, Tài Pham, Tommaso Mauri, Elena Spinelli, Giacomo Grasselli, Savino Spadaro, Carlo Alberto Volta, Francesco Mojoli, Dimitris Georgopoulos, Eumorfia Kondili, Stella Soundoulounaki, Tobias Becher, Norbert Weiler, Dirk Schaedler, Oriol Roca, Manel Santafe, Jordi Mancebo, Nuria Rodríguez, Leo Heunks, Heder de Vries, Chang-Wen Chen, Jian-Xin Zhou, Guang-Qiang Chen, Nuttapol Rit-tayamai, Norberto Tiribelli, Sebastian Fredes, Ricard Mellado Artigas, Carlos Ferrando Ortolá, François Beloncle, Alain Mercat, Jean-Michel Arnal, Jean-Luc Diehl, Alexandre Demoule, Martin Dres, Quentin Fossé, Sébastien Jochmans, Jonathan Chelly, Nicolas Terzi, Claude Guérin, E. Baedorf Kassis, Jeremy Beitler, Davide Chiumello, Erica Ferrari Luca Bol-giaghi, Arnaud W. Thille, Rémi Coudroy, Laurent Papazian

**Affiliations:** 1 Critical Care Department, Parc Taulí Hospital Universitari, Institut d’Investigació I Innovació Parc Taulí (I3PT-CERCA),, Carrer Parc Taulí, 1, 08208 Sabadell, Spain; 2 Centro Investigación Biomédica en Red de Enfermedades Respiratorias (CIBERES), Instituto de Salud Carlos III, Madrid, Spain; 3 Institut d’Investigació i Innovació Parc Taulí (I3PT-CERCA), Sabadell, Spain; 4 Keenan Research Center for Biomedical Science, Li Ka Shing Knowledge Institute, Unity Health Toronto, Toronto, ON Canada; 5 Interdepartmental Division of Critical Care Medicine, University of Toronto, Toronto, ON Canada; 6 Division of Respirology, Department of Medicine, University Health Network and Sinai Health System, Toronto, ON Canada; 7 Critial Care Department, Althaia Xarxa Assistencial Universtaria de Manresa, Manresa, Spain; 8 IRIS - Catalunya Central I Grup de Recerca de Malalt Crític, Manresa, Spain; 9 Critical Care Department, Hospital Británico, Buenos Aires, Argentina; 10 Unidad de Cuidados Intensivos Cardiológicos, Hospital Universitario Central de Asturias. Universidad de Oviedo, Oviedo, Spain; 11 Centro de Investigación Biomédica en Red de Salud Mental (CIBERSAM), Instituto de Salud Carlos III, Madrid, Spain; 12 Departament de Medicina, Universitat Autònoma de Barcelona, Bellaterra, Spain; 13 Service de Médecine Intensive-Réanimation, Hôpital de Bicêtre, DMU CORREVE, FHU SEPSIS, Groupe de Recherche Clinique CARMAS, Université Paris-Saclay, AP-HP, Le Kremlin-Bicêtre, France; 14 Université Paris-Saclay, UVSQ, Univ. Paris-Sud, Inserm U1018, Equipe d’Epidémiologie Respiratoire Intégrative, Center de Recherche en Epidémiologie et Santé Des Populations, Villejuif, France; 15 Better Care, SL, Sabadell, Spain

**Keywords:** Airway pressure deformation, Flow starvation, Patient–ventilator interaction, Asynchronies, Artificial intelligence algorithms

## Abstract

**Background:**

Flow starvation is a type of patient-ventilator asynchrony that occurs when gas delivery does not fully meet the patients’ ventilatory demand due to an insufficient airflow and/or a high inspiratory effort, and it is usually identified by visual inspection of airway pressure waveform. Clinical diagnosis is cumbersome and prone to underdiagnosis, being an opportunity for artificial intelligence. Our objective is to develop a supervised artificial intelligence algorithm for identifying airway pressure deformation during square-flow assisted ventilation and patient-triggered breaths.

**Methods:**

Multicenter, observational study. Adult critically ill patients under mechanical ventilation > 24 h on square-flow assisted ventilation were included. As the reference, 5 intensive care experts classified airway pressure deformation severity. Convolutional neural network and recurrent neural network models were trained and evaluated using accuracy, precision, recall and F1 score. In a subgroup of patients with esophageal pressure measurement (Δ*P*_es_), we analyzed the association between the intensity of the inspiratory effort and the airway pressure deformation.

**Results:**

6428 breaths from 28 patients were analyzed, 42% were classified as having normal-mild, 23% moderate, and 34% severe airway pressure deformation. The accuracy of recurrent neural network algorithm and convolutional neural network were 87.9% [87.6–88.3], and 86.8% [86.6–87.4], respectively. Double triggering appeared in 8.8% of breaths, always in the presence of severe airway pressure deformation. The subgroup analysis demonstrated that 74.4% of breaths classified as severe airway pressure deformation had a Δ*P*_es_ > 10 cmH_2_O and 37.2% a Δ*P*_es_ > 15 cmH_2_O.

**Conclusions:**

Recurrent neural network model appears excellent to identify airway pressure deformation due to flow starvation. It could be used as a real-time, 24-h bedside monitoring tool to minimize unrecognized periods of inappropriate patient-ventilator interaction.

**Supplementary Information:**

The online version contains supplementary material available at 10.1186/s13054-024-04845-y.

## Background

In critically ill patients under invasive mechanical ventilation (IMV) on square-flow assisted ventilation, visual inspection of the ventilator waveforms allows the detection of patient-ventilator asynchronies. During inspiration, the depression or deformation of the airway pressure (*P*_aw_) waveform from the expected passive profile reflects flow starvation [1]. Flow starvation is a type of patient-ventilator asynchrony that occurs when gas delivery does not fully meet the patients’ ventilatory demand due to an insufficient airflow and/or a high inspiratory effort [2, 3]. Flow starvation leads to an additional load on patients and an elevated energy consumption by the respiratory muscles that can cause patient self-inflicted lung injury and concentric load-induced diaphragm injury [4, 5] due to increased transpulmonary pressures, lung strain and stress. Moreover, insufficient airflow produces dyspnea, particularly air hunger which is the most distressing type of dyspnea [6], and could induce harmful asynchronies like double triggering [7, 8]. Air hunger and dyspnea cause patient discomfort, increase anxiety, often leading to higher sedative doses, promoting delirium, and increased duration of IMV, intensive care unit (ICU) and hospital stay [9, 10].

The identification of abnormal patterns of *P*_aw_ waveform at the bedside by visual inspection of the ventilator requires extensive knowledge of respiratory physiology, and is limited for short time periods of observation, leading to massive underdiagnosis [11]. Frequently, these anomalous patterns can be managed by adjusting the ventilator [12]. Automatic methods to continuously identify flow starvation through the identification of *P*_aw_ waveform deformation could warn clinicians to modify the ventilator settings to limit discomfort and to minimize the development of potentially injurious asynchronies.

The aim of this study was to develop a supervised artificial intelligence (AI) algorithm for continuous identification and classification of *P*_aw_ waveform deformation patterns in patient-triggered breaths, on square-flow assisted ventilation caused by a mismatch between the patient’s ventilatory demands and ventilator’s support. Additionally, we aimed to explore the association between the pattern of *P*_aw_ deformation and the inspiratory effort evaluated by the esophageal pressure (*P*_es_.)

## Methods

### Design

Ancillary analysis of two prospective cohort studies in adult critically ill patients receiving IMV. Patients admitted to the ICU (St. Michael's Hospital (Toronto, Canada) and Parc Taulí Hospital Universitari (Sabadell, Spain) receiving IMV > 24 h on square-flow assisted ventilation were included. Patients or their surrogate decision-makers provided informed consent to participate in the study collecting waveforms for processing and analysis.

### Data collection

The data from St. Michael’s Hospital was part of the BEARDS study (NCT03447288) and included ventilator waveforms (airflow and *P*_aw_) and *P*_es_ from the first 7 days of IMV [13]. The data from Parc Taulí Hospital Universitari included ventilatory waveforms (airflow and *P*_aw_), from IMV patients, continuously recorded using the Better Care system (BCLink, Better Care, Sabadell, Spain. US patent No. 12/538,940) proceeding from several studies on patient-ventilator asynchronies (NCT02390024, NCT02714751, NCT03451461 and NCT05363332) from intubation to IMV liberation [17, 18]. Signals were pre-processed by MATLAB (The MathWorks, Inc., vR2018b, Natick, MA, USA). BEARDS signals were filtered with a Butterworth low-pass filter at 15 Hz to remove noise. All signals were decimated at a sampling rate of 40 Hz.

Two investigators (LS and VSP) with expertise in signal processing of ventilator waveforms visually inspected the tracings and selected breaths for the analysis. Eligible tracings were those: (1) with patient-triggered breaths, and (2) on square-flow volume assist-control ventilation. From those tracings, two subgroups of breaths were pre-selected. The subgroup 1 without inspiratory phase deformation, and the subgroup 2 with variable degree of deformation in the inspiratory phase on the *P*_aw_ waveform as compared to normal breaths. Additionally, breaths were selected to have a balanced sampling at the beginning of IMV, in intermediate period and at the end of IMV. Finally, a sample of 6500 breaths of them were selected initially, and was estimated post-hoc based on the learning curve.

Exploration of the association between the pattern of *P*_aw_ deformation and the inspiratory effort evaluated with the delta of *P*_es_ (Δ*P*_es_) was performed only in the subgroup of patients of the BEARDS study with esophageal pressure tracings.

### *Experts’ annotation of P*_*aw*_* deformation severity*

**Fig. 1:**
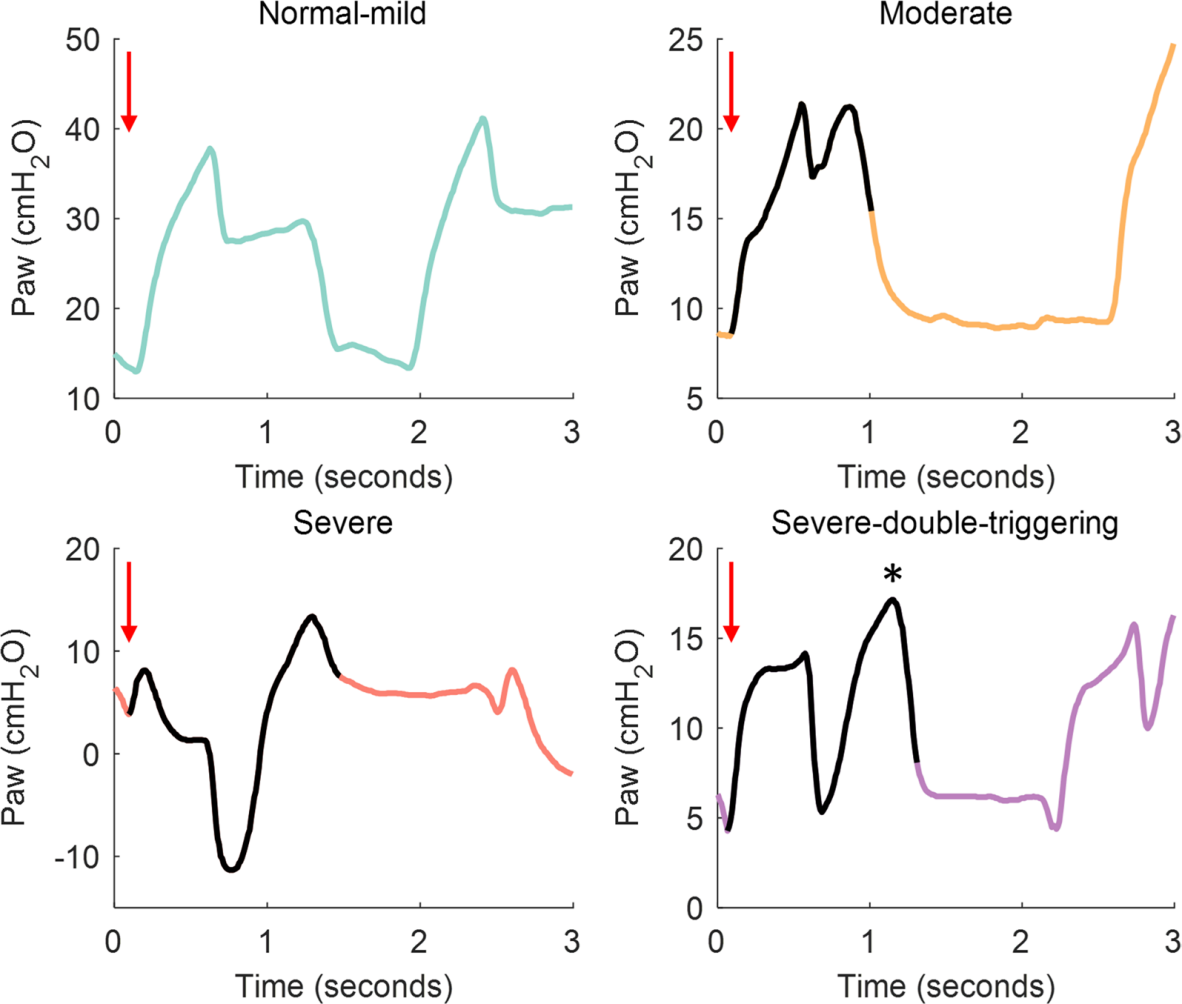
Representative examples of the airway pressure (*P*_aw_) deformation patterns classification on the pressure–time waveform. Red arrows show the initiation of the patient-triggered breath. The *P*_aw_ deformation in the moderate, severe and severe with double triggering tracings is represented by a solid black line on the *P*_aw_ tracings. The asterisk shows the second breath added to the first one in the severe breaths with double triggering (breath stacking)

The selected ventilator tracings (*P*_aw_ and flow) were visually inspected by five ICU senior physicians (LlB, RF, GMA, GM, CDH), with extensive clinical experience in IMV and management of asynchronies. They classified all breaths by identifying the amount of *P*_aw_ deformation patterns as compared to a passive insufflation, which were stored in an interactive web application specifically developed for this purpose (Additional file 1: additional details in online data supplement Figure E1). *P*_aw_ deformations were classified by the researchers in one of 3 pattern categories: normal (or with mild deformation), moderate deformation and severe deformation (Fig. 1). Agreement between researchers about the classification of *P*_aw_ deformation was determined with the majority voting method (three of five experts agreement) [14]. In case of disagreement between the experts, the breaths were re-analyzed by the senior coordinator (LlB) who decided whether the breaths were included or not in the analysis. Breaths were excluded from analysis when: (1) 2 of the 5 annotators noted deemed them wrong/confusing (i.e., technical issues), and (2) the following annotation pattern was present: 2 votes normal-mild, 1 vote moderate and 2 votes severe. The percentage of patients in each category can be found in online data supplement.

Double-triggering breaths were identified from the tracings (through a validated algorithm in the cohort of patients from Parc Taulí Hospital Universitari and visually in the cohort of patients from St. Michael’s Hospital), and were considered as a separate category in order to investigate their incidence.

### *Algorithms for detection of P*_*aw*_* deformation*

The expert classification was used for training independently two machine learning models for automatically classifying the *P*_aw_ deformation patterns: recurrent neural network and convolutional neural network. The algorithms' input data consisted of the inspiratory phase of *P*_aw_ waveforms, which were resampled to 80 samples to ensure that all breaths have the same length. The goal was to detect P_aw_ deformation during the inspiratory phase of patient-triggered breaths in square-flow volume assist-control ventilation.

The recurrent neural network algorithm is appropriate for long-sequence applications, since their architecture is designed to predict an output for each element [15, 16]. In particular, for time series, the most commonly used type is the long short-term memory [17, 18], that learns from long-term dependencies. In this study, two hidden layers of 128 neurons were used and a fully connected layer was added at the end of the long short-term memory to classify into one of the three categories. The convolutional neural network algorithm using a 1D convolution (1D convolutional neural network) contains convolution kernels/filters that can be interpreted as a time series application. These kernels move in a single time direction from the beginning of a time series toward its end, performing the convolution. One application behind the use of multiple filters is the ability to learn multiple discriminative features useful for the classification task [16]. Once the models have learned the different patterns the time required to detect a pattern for both algorithms is very similar. Additional information on the implemented models can be found in the online data supplement (Additional file 1: Figures E2 and E3). Models were implemented using Python (v 3.9.7) with the PyTorch (v. 1.11.0) package and run on a desktop computer (Windows 10 Pro 64-bit, Intel(R) Core(TM) i7-6700 CPU @ 3.40 GHz and 16 GB RAM).

### Statistics

Agreement between researchers about the classification of *P*_aw_ deformation was determined as the percentage of breaths with agreement (three of five experts) considering the majority voting method [14] and the Fleiss’ kappa coefficient. The recurrent neural network and convolutional neural network models were trained using the repeated holdout cross-validation method. The dataset was divided into an 80–20 train-validation split, with 80% of the data used for training and 20% for validation. This process was repeated 15 times, with each repetition using a different randomly selected subset for validation. Subsequently, median values were derived from the outcomes of each validation step, enhancing a more robust estimate of the model's performance. Performance measures of AI algorithms (accuracy, recall, F-1 score and precision) were used to measure the effectiveness of the algorithms (Additional file 1: additional information on the online data supplement). To ensure an heterogeneous dataset and a good performance of the model, we have lumped together the data from both centers. Wilcoxon signed-rank test was used to investigate the relationship between the patterns of *P*_aw_ deformation and inspiratory time (*T*_i_) and inspiratory peak airflow. Bonferroni correction (*α* = 0.05/6 = 0.0083) was considered. We analyzed learning curves of applied models to examine sample size. Further details, including a comparison of the sample size to the success rate, can be found in the online data supplement (see Additional file 1: Figure E4).

## Results

**Fig. 2:**
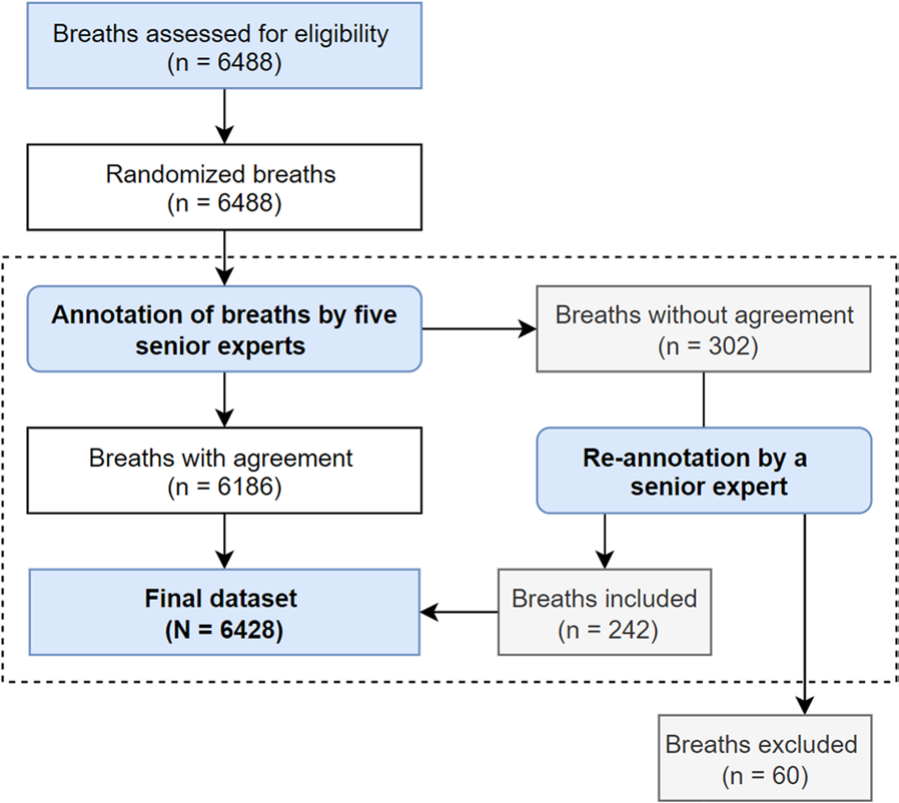
Flowchart of the breath annotation procedure from ventilator tracings

Table 1 shows the patient’s characteristics (data were expressed as median [interquartile range]). A total of 6488 breaths from 28 patients receiving IMV were classified by experts: 559 from St. Michael's Hospital and 5929 from Parc Taulí Hospital Universitari (Fig. 2). Of these, in 302 breaths (4.6%) the experts disagree and were re-analyzed; among these, 60 breaths were finally excluded. Therefore, the final dataset included 6428 breaths classified by experts as follows: 2708 normal-mild (42.1%), 1535 moderate (23.8%), and 2185 severe *P*_aw_ deformation (33.9%). The inter-expert agreement was 95.4% (Additional file 1: additional information in the online data supplement and Figure E5).

**Table 1:** Patients’ demographic and clinical characteristics at admission

Patients' demographic and clinical characteristics at admission	*n* = 28
Age	63 [57–70]
Female (%)	4 (14%)
Reason for MV, *n* (%)	
*Pneumonia*	11 (39%)
*Sepsis*	3 (11%)
*COVID-19*	10 (36%)
*Other causes*	4 (14%)
APACHE II at admission	14 [11–23]
SOFA	7 [5–9.2]
Median duration of MV (range), in days	17 [13–26]
Median ICU–LOS (range), in days	23.5 [16–34.8]
Median hospital–LOS (range), in days	39 [23–63.5]
ICU mortality (%)	5 (18%)

Data are represented as median [25th, 75th percentiles] or percentages. Definition of abbreviations: APACHE II: Acute Physiology and Chronic Health Evaluation. ICU: Intensive care unit. LOS: length of stay. MV: mechanical ventilation

**Fig. 3:**
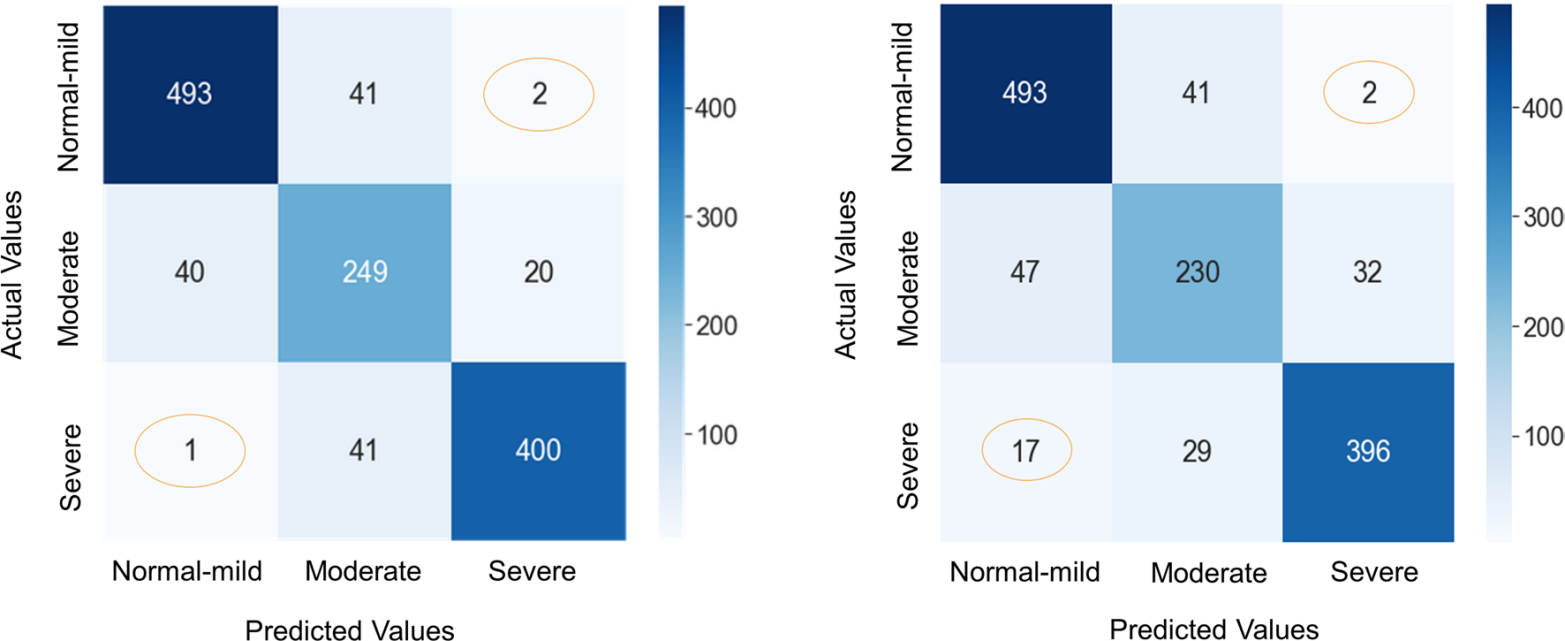
Confusion matrix for the recurrent neural network (RNN) and convolutional neural network (CNN) validation processes, respectively. The implemented models provide a strong performance for normal-mild and severe patterns. The reported performance metrics are the average across the 15 repetitions

The validation dataset consisted of 1287 breaths including 536 normal-mild (41.7%), 309 moderate (24.0%), and 442 severe *P*_aw_ deformation (34.4%). The confusion matrix (Fig. 3) shows the breakdown of the classification provided by the machine learning classifiers compared to the human expert labels for the validation phase. The recurrent neural network algorithm accurately classified 92% of normal-mild (493/536), 80.6% of moderate (249/309), and 90.5% of severe (400/442) *P*_aw_ deformation, and 145 breaths of total validation dataset (11.3%) were misclassified. The recurrent neural network algorithm performed very well at the extremes (severe vs. normal-mild), as it labeled only one severe breath as normal-mild and two normal breaths as severe. Overall, the recurrent neural network performance had 87.9% [87.6–88.3] accuracy, 87.7% [87.5–88.2] precision, 87.9% [87.6–88.3] recall and 87.7% [87.4–88.1] F1 score. The convolutional neural network algorithm accurately classified 92% of normal-mild (493/536), 74.4% of moderate (230/309), and 89.6% of severe (396/442) *P*_aw_ deformation, and 168 breaths (13.1%) were misclassified. Again, error between the extremes (severe vs. normal-mild) were negligible: 2 normal-mild breaths were classified as severe, and 17 severe breaths were classified as normal-mild. Overall, the convolutional neural network performance was 86.8% [86.6–87.4] accuracy, 87% [86.7–87.3] precision, 86.8% [86.6–87.4] recall and 86.9% [86.6–87.3] F1 score. (Additional file 1: Table E1 in online data supplement shows details of performance metrics obtained during the training and validation process for the 15 times models were trained.)

Median ventilator inspiratory time, peak inspiratory airflow, respiratory rate, positive end expiratory pressure (PEEP) and expiratory time were similar between the breaths corresponding to the 3 groups of *P*_aw_ deformation. Tidal volume was lower in the most severe patterns, with no statistically significant differences (Additional file 1: Figure E6 and Table E2 in the online data supplement). Double triggering was only present in breaths with severe *P*_aw_ deformation (8.8% of breaths with severe deformation).

**Fig. 4:**
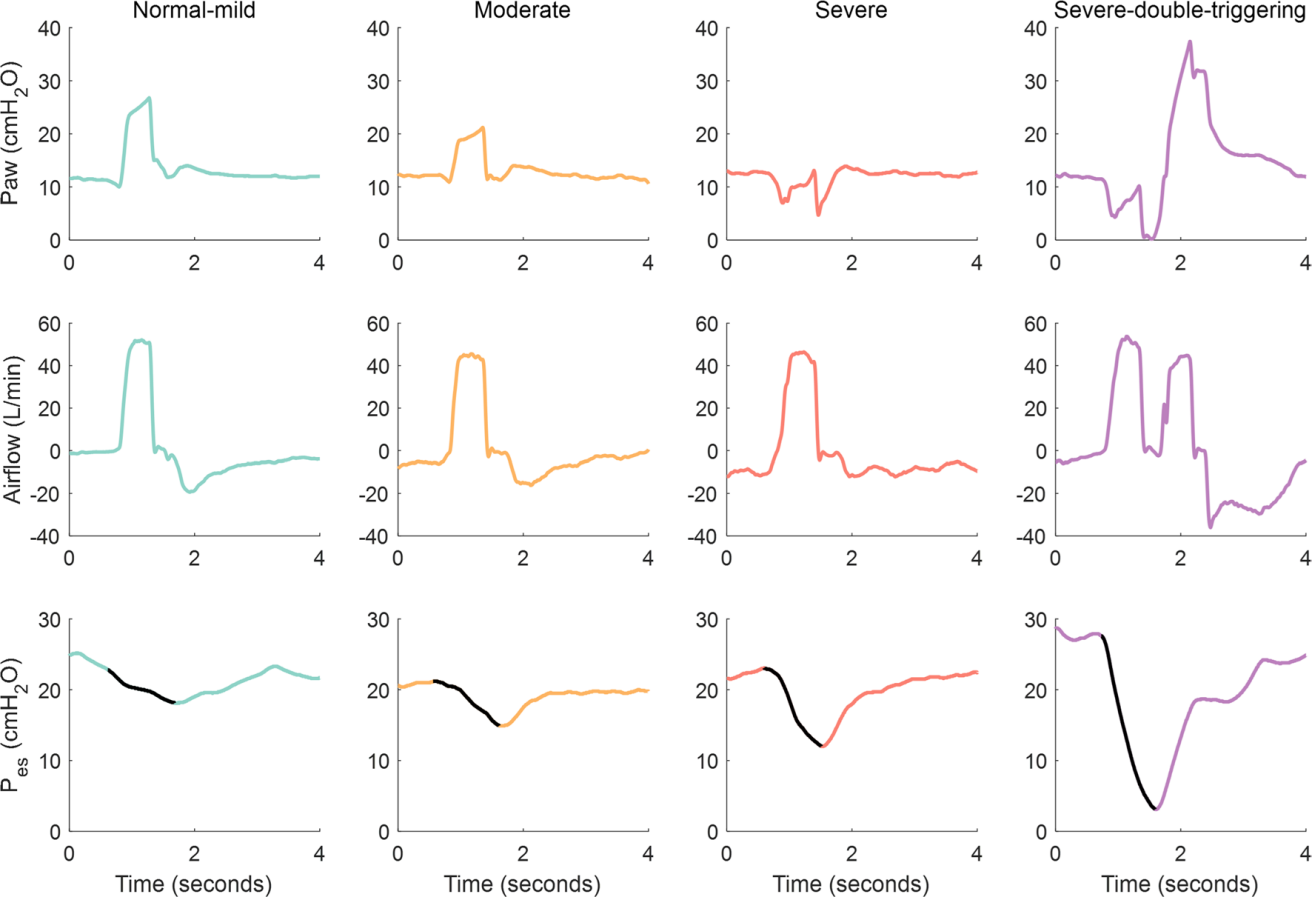
Representative examples of airway pressure (*P*_aw_), airflow and esophageal pressure (*P*_es_) tracings during square-flow assisted control ventilation corresponding to normal-mild breath, moderate breath, severe breath and double triggering, respectively. The esophageal swing is represented by solid black lines on the *P*_es_ tracings, which increases in relation to the different patterns (the greater the swing, the greater the inspiratory effort)

In the secondary analysis of BEARDS patients with esophageal pressure measurements Δ*P*_es_ was > 8 cmH_2_O in 2.4%, 35.4%, and 94.8% of breaths with normal-mild, moderate or severe *P*_aw_ deformation, respectively, whereas Δ*P*_es_ was > 10 cmH_2_O in 74.4% of breaths with severe *P*_aw_ deformation (Additional file 1: Additional information in Table E3 online data supplement). Figure 4 shows representative examples of *P*_aw_, airflow and *P*_es_ tracings corresponding to breaths of different severity.

## Discussion

The main findings of this study are: (1) AI models can detect and classify breath-by-breath *P*_aw_ deformation patterns with high accuracy; (2) breaths classified as having severe *P*_aw_ deformation exhibit stronger inspiratory efforts; (3) double triggering only occurs in breaths with severe *P*_aw_ deformation.

A major goal of IMV is to unload the respiratory muscles to avoid exhaustion while avoiding muscle atrophy [19, 20]. However, during clinical situations of high inspiratory demands or insufficient delivered airflow, patients may develop strong inspiratory efforts [21]. This may be associated with dyspnea and both patient self-induced lung injury and myotrauma [22, 23]. In square-flow volume assist-control ventilation, sometimes the patient triggers the ventilator by slightly lowering *P*_aw_, followed by the mechanical insufflation that intends to reduce the work of breathing [20]. The muscular pressure could be estimated by the difference in *P*_aw_ between passive and active circumstances. The greater drop in the *P*_aw_ waveform during insufflation, the greater inspiratory effort of the patient [12, 24, 25]. Although the *P*_aw_ waveform can be quickly examined during square-flow volume assist-control ventilation to identify a significant deformation [26], underdiagnosis is frequent, either because of failure to recognize the deformation or because professionals can only inspect waveforms for short time periods [11].

Convolutional neural network and recurrent neural network models have shown the best results on automatically detecting patient-ventilator asynchronies e.g., double triggering, ineffective effort, delayed cycling and premature cycling [15, 17, 18, 27–30]. Convolutional neural network algorithms detected different types of patient-ventilator asynchronies with an accuracy ranging from 97 to 99% [15, 17, 18, 27–30] whereas recurrent neural networks, in particular long short-term memory, performed slightly lower results between 91 and 98.3% [15]. In the present study, two different neural networks have been implemented, a long short-term memory and a 1D convolutional neural network. Convolutional neural networks are currently considered the most advanced models due to their best results in patient-ventilator asynchronies detection, but in our study, the recurrent neural network model showed similar accuracy. One explanation may be that recurrent neural networks are also suited to handle time-dependent sequences or data [15]. These networks use time series information to identify patterns between input and output. The memory of recurrent neural network algorithms allows them to learn more about the long-term dependencies of the data and understand the full context of the sequence when making the next prediction [15, 31].

Currently, the gold standard for the identification and quantification of strong inspiratory efforts is the measurement of *P*_es_ swing. However, it is not commonly used due to its complexity and invasiveness [32–34]. Similarly to our study, Telias et al. [34] have recently developed an automated algorithm based on *P*_es_ measurements that accurately generates and quantifies the muscular pressure for synchronous and dyssynchronous inspiratory efforts. They suggest that those patients with strong efforts detected by the algorithm might benefit from *P*_es_ monitoring. In recent years, several continuous monitoring systems that integrate signals in real-time have emerged and, through the application of validated algorithms, can automatically and continuously identify asynchronies [13, 35–38]. In the present study, a high percentage of breaths classified as severe exhibit Δ*P*_es_ > 8 or 10 cmH_2_O, suggesting that *P*_aw_ deformation is frequently associated with strong muscular efforts.

Double triggering was present exclusively in breaths with a severe *P*_aw_ deformation (8.8% of them) [34]. Double triggering is one of the most potentially injurious patient-ventilator asynchronies in assisted volume-controlled ventilation, due to the high *P*_aw_ and very high tidal volume resulting from the accumulation of two consecutive breaths [39–41]. This can generate higher transpulmonary and transvascular pressure gradients, increasing tissue stress and strain, and resulting in an unequal pressure distribution in lung-dependent areas [42], which can favor ventilator-induced lung injury [43, 44]. Among the factors associated with the development of double triggering, short ventilator inspiratory time and/or low airflow setting have also been recognized as important [41].

Our study make a significant contribution to the field of patient-ventilator asynchrony detection. Firstly, it introduces an innovative solution for classifying flow starvation during square-flow assisted ventilation using convolutional neural network and recurrent neural network models. The majority of existing patient-ventilator asynchrony algorithms [37, 45] primarily focus on identifying common forms of asynchronies such as double triggering, ineffective effort, and short- and prolonged cycling. In contrast to previous studies [27–30] employing a binary classification for asynchrony classification, our work adopts a multiclass approach. This approach enables clinicians to differentiate, for instance, between moderate and severe degrees of Paw deformation. Secondly, our dataset construction strategy, which incorporates waveforms from two different medical centers, allows us to assess the extrapolation capability of deep learning methods. To ensure a balanced representation and prevent overemphasis on specific patients, the number of breaths per patient in each class was capped at a maximum of 350 breaths. Additionally, breaths were selected to create a balanced sample across the initial, intermediate, and final stages of IMV. Thirdly, the architectural design of our implemented models utilizes a single branch corresponding to the inspiratory phase of the Paw waveform, with a fixed size of 80 sample points as input to the tensor. This results in models of lower complexity compared to other studies [27, 30] that employ deep learning approaches for the classification of patient-ventilator asynchronies. Lastly, our work presents an automated algorithm for detecting flow starvation, aiming to improve the underdiagnosis of patient-ventilator asynchronies by visual examination of ventilator waveforms at the bedside [11, 46]. The AI model could provide an accurate classification of breaths with severe *P*_aw_ deformation, based on the analysis of *P*_aw_ waveform. Therefore, the continuous assessment of *P*_aw_ deformation by using AI technologies could alert clinicians about the presence of excessively high inspiratory efforts or associated with insufficient airflow.

This study has limitations. First, the deep learning model was only applied to IMV under square-flow assisted ventilation, but it is one of the most widely used mode of ventilation [47, 48]. Our AI model stands as an initial technological approach that needs further evaluation and implementation with additional data and other ventilator modes to enhance its robustness and generability. Currently the ventilators do not provide alarm systems to notify the presence of abnormal *P*_aw_ waveforms patterns. From a clinical perspective, computerized systems are needed to connect and agnostically interoperate ventilator waveforms. A continuous analysis of *P*_aw_ waveforms using AI models could potentially be integrated into ICU mechanical ventilators or monitoring centers, providing valuable support and alert tool for clinicians [49–51]. Second, the recurrent neural network and convolutional neural network models need to be trained with sufficient data [52], and although our sample of about 6500 breaths may appear small, it has yielded very good performance on the training and validation datasets. Higher large-scale labeling efforts are costly and time-consuming, and often require extensive domain knowledge or technical expertise to implement a particular medical task, often resulting in large-scale inefficiencies in clinical AI workflows. Furthermore, these methods can only predict events on which they have been trained, which restricts their widespread applicability. Therefore, these label learning methods may not be as powerful in environments where access to a diverse set of high-quality data is limited [53].

## Conclusions

Our study shows that AI, in particular recurrent neural networks, could be an excellent tool to identify airway pressure deformation associated to strong inspiratory efforts during square-flow volume assist-control ventilation, allowing to minimize unrecognized periods of abnormal and potentially injurious patient-ventilator interaction.

### Supplementary Information

**Additional file 1**. Supplementary materials, tables and figures.

## Data Availability

The datasets used and analyzed during this study are available from the corresponding author on reasonable request.

## Abbreviations

AI: Artificial intellligence
cmH_2_O: Centimeters of water
ICU: Intensive Care Unit
IMV: Invasive mechanical ventilation
*P*_aw_: Airway pressure
PEEP: Positive end expiratory pressure
*P*_es_: Esophageal pressure
*T*_i_: Inspiratory time
