# Supplementary material for: Flow starvation during square-flow assisted ventilation detected by supervised deep learning techniques
Source: Crit Care. 2024 Mar 14;28:75. doi: 10.1186/s13054-024-04845-y (PMC10938655; doi:10.1186/s13054-024-04845-y)
Supplement: Supplementary file 1 — Additional file 1. Supplementary materials, tables and figures. [file 13054_2024_4845_MOESM1_ESM.docx]

**Flow starvation during square-flow assisted ventilation detected by supervised deep learning techniques**

**Online Data Supplement**

Candelaria de Haro, Verónica Santos-Pulpón , Irene Telías, Alba Xifra-Porxas, Carles Subirà, Montserrat Batlle, Rafael Fernández, Gastón Murias, Guillermo Muñiz-Albaiceta, Sol Fernández-Gonzalo, Marta Godoy-González, Gemma Gomà, Sara Nogales, Oriol Roca, Tai Pham, Josefina López-Aguilar, Rudys Magrans, Laurent Brochard, Lluís Blanch, Leonardo Sarlabous and the BEARDS study investigators

**Interactive web application developed for breath annotation**

In this study we developed an interactive web application to classify breaths into one of three P_aw_ waveform deformation patterns (Figure E1). The interactive web application was developed using Shiny, a free and open source R package. The app was configured to display two-second airflow and airway pressure tracings. Tracings were loaded from csv files. It also had a slider to select the index of the breath to be displayed and buttons to move forward and backward through the breaths. The user could select the annotator's name from a dropdown list, and there were buttons to assign labels to the data (normal/mild, moderate and severe). In addition, a button to discard erroneous breaths was included (wrong/confusing). The labels were saved in a PostgreSQL database table. When the user clicked one of the label buttons, the label value (0, 1, 2, or 3) was inserted into the database table with the annotator's name and the cycle index. Subsequently, the cycle index was incremented, and the next cycle's data was displayed. Finally, the application also displayed the previous annotation assigned to the cycle when the user clicked the "Show Previous Annotation" button.

Figure E1. Interactive web application (Shiny app) developed for breath annotation. Shiny is a package that makes it easy to build interactive web apps straight from R.


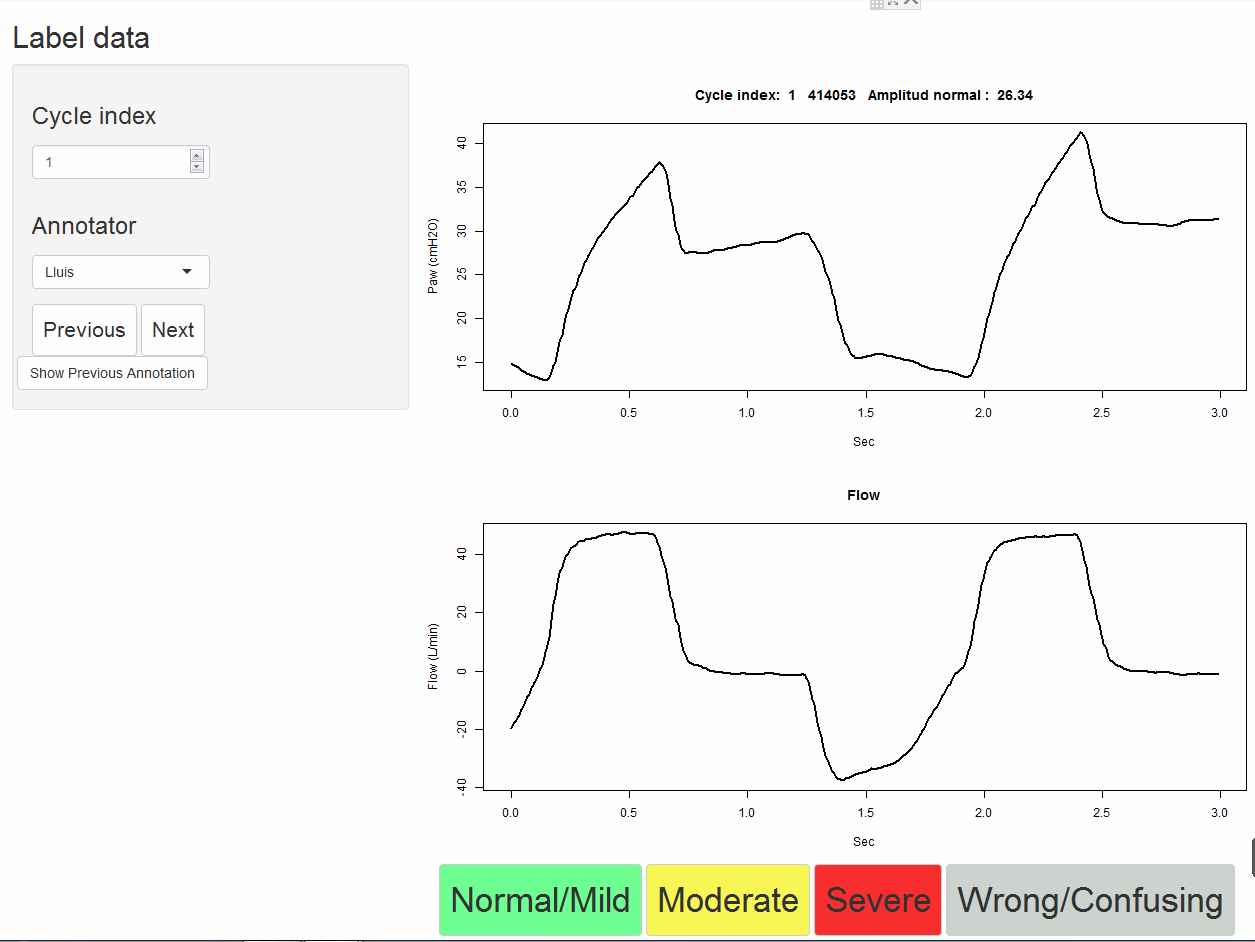


**Dataset distribution**

It is important to have balanced datasets when training artificial intelligence (AI) models because imbalance in the distribution of labels or classes in the data can lead to inaccurate or biased results. When the dataset is not balanced, AI models may learn inappropriate patterns and produce results that favour the most frequent classes in the training data (1) . This is because AI models are designed to minimise error in the training data, meaning that they focus on correctly classifying the most common classes and may ignore or underperform for less common classes. This problem is particularly problematic in applications where accurate classification of all classes is crucial, such as in disease detection or fraud identification. If less common classes are incorrectly classified, this can have serious consequences (2).

To mitigate this problem, it is important to have balanced datasets when training AI models. This can be achieved in several ways, such as collecting more data for the less common classes, sub-sampling the data for the most frequent classes, or using synthetic data generation techniques. By balancing the classes in the dataset, AI models can learn more accurate and balanced patterns, resulting in better performance across classes. In short, having a balanced dataset is critical to ensure accuracy and fairness in the results of AI models.

Our final dataset consists of 6,428 breaths. The contribution of each patient to the dataset in each category was conditional on the availability of breaths with high potential to be classified by the experts in one of the three categories. The maximum number of cycles a patient can contribute to each category was limited to 350 cycles. Therefore, not all patients contributed equally to the dataset. Of the 28 patients involved in the study, 67.9% (19 patients) contributed breaths to all three categories, 25% (7 patients) contributed in two categories (normal-mild and moderate: 4, moderate and severe: 2 and normal-mild and severe: 1) and 7.1% (2 patients) contributed in only one category (normal-mild or severe, respectively).

**Models architecture**

**Recurrent Neural Networks**

Recurrent Neural Networks (RNNs) are a type of neural network designed to handle sequential data, such as time series (3). Unlike traditional neural networks, which only process fixed-size inputs and produce fixed-size outputs, RNNs can take inputs of variable length and generate variable-length outputs as well. This feature makes them well-suited to time series analysis, where the length of input and output sequences can vary (4). RNNs work by maintaining an internal state, which is updated at each time step as new inputs are processed. This enables them to capture the temporal dependencies between different elements of the input sequence.

However, RNNs can suffer from the problem of vanishing gradients (5), which can make it challenging to train them on long sequences. Long Short-Term Memory (LSTM) (6) networks are a type of RNN that address this problem. They use memory cells and gating mechanisms to regulate the flow of information through the network, allowing them to learn long-term dependencies in the input sequence. The memory cells store information over longer periods, while the gating mechanisms control the flow of information into and out of the cells. This enables LSTM networks to capture both short-term and long-term dependencies in the input data. Both, RNNs and LSTM networks are powerful tools for analysing time series data in the healthcare field (7,8). They can be used for a variety of applications, such as predicting disease progression or patient outcomes, analysing medical images, and extracting insights from electronic health records (9).

Figure E2 shows the implemented RNN. The first layer of the network is the input layer, which takes in the input data and prepares it for processing. The input layer feeds the data to the next layer, which is the hidden layer. The hidden layer is composed of two LSTMs of 128 neurons each, which have their own internal memory that can hold information for a long period of time. This allows the network to capture long-term dependencies in the input data. The output from the hidden layer is then passed to the next layer, which is the classification layer. The classification layer applies a set of weights to the output of the hidden layer to classify the input data into one of several categories. Finally, the output from the classification layer is passed to the output layer, which produces the final output of the network.


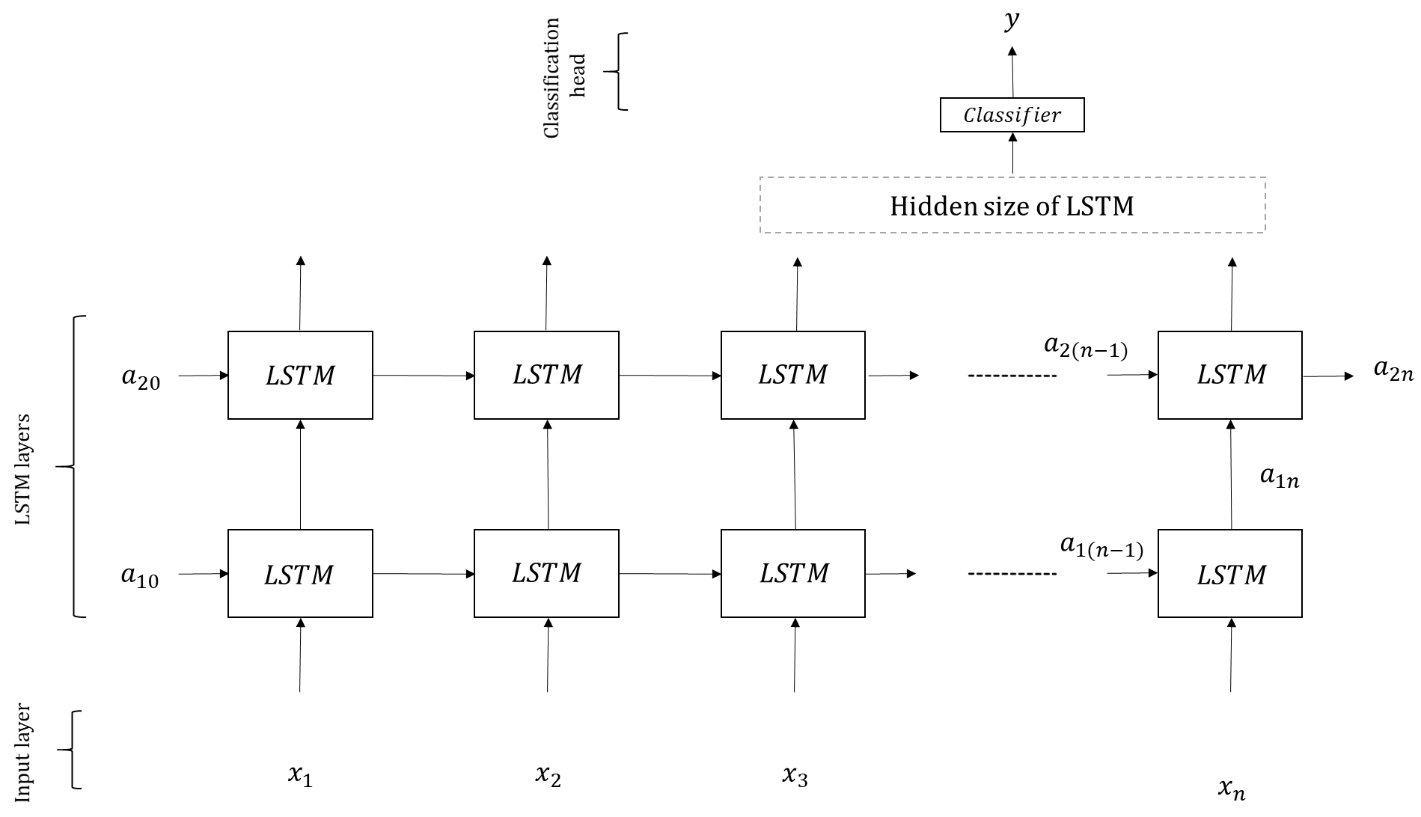


Figure E2. Implemented Recurrent Neural Network.

**Convolutional Neural Networks**

Convolutional Neural Networks (CNN) are a type of deep learning algorithm that is primarily used in computer vision tasks such as image and video recognition (10,11). They have also been successfully applied to time series data, particularly in the field of healthcare to improve disease diagnosis (12), risk prediction (13), and drug discovery (14).Time series data refers to data that is collected over time, such as vital signs or sensor data from medical devices. CNNs are able to extract features from this data by convolving filters over the input data and performing a non-linear transformation to produce a new output feature map. This allows CNNs to capture temporal patterns in the data that would be difficult to detect using traditional statistical methods (15).

Figure E3 shows the implemented CNN. In this network, we will use 1D convolution as it contains convolution kernels/filters that can be interpreted as the application and sliding of filters over the time series. These kernels move in a single time direction, from the beginning of the time series towards its end, performing the convolution. The filters can also be viewed as a generic non-linear transformation of a time series. Therefore, more than one filter can be applied to the time series, resulting in a multivariate time series whose dimensions are equal to the number of filters used. One benefit of using multiple filters on an input time series is the ability to learn multiple discriminative features useful for the classification task (16,17). Following the convolutional layer, a Batch Normalization layer is applied to help the network converge quickly, followed by a Rectified Linear Unit (ReLU), which is an activation function. Finally, a Max Pooling layer is used to downsample the obtained result (16). These blocks can be applied several times to make the model more complex and obtain deeper features.

Specifically, we consider four convolutional blocks for this task. To classify the data, the convolutional blocks must be followed by a discriminative classifier. The last discriminative layer takes as input the flattened convolutions' result and outputs a probability distribution over the three designated classes. This layer is followed by a softmax operation.


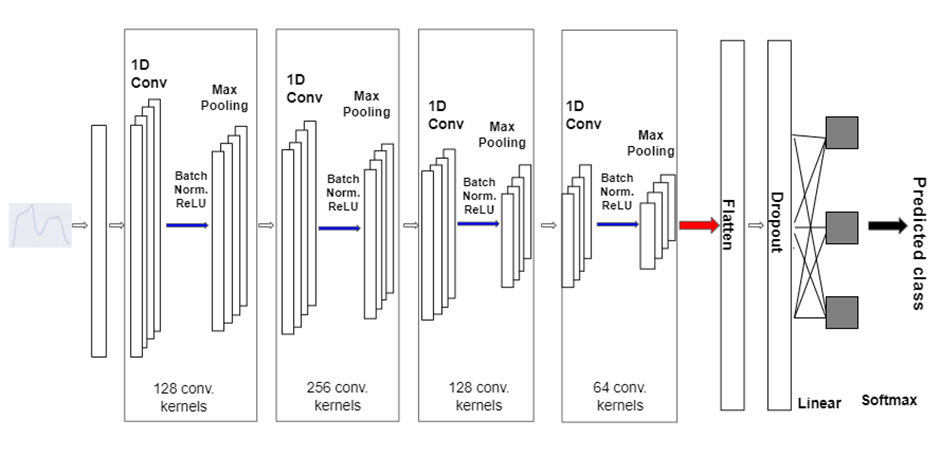


Figure E3. Implemented Convolutional Neural Networks.

**Performance metrics**

Performance metrics were collected from implemented models, and the training and rest procedures were iterated 15 times. This iterative process is valuable for obtaining robust and reliable performance evaluations. It allows for a more comprehensive understanding of how model generalizes across different subsets of the data. By performing these iterations, you can observe the consistency and stability of the model’s performance metrics, which is crucial in assessing its reliability in real-world scenarios.

Performance measures of AI algorithms (accuracy, recall, F-1 score and precision) were used to measure the effectiveness of the algorithms (20):

Precision (macro average):

$\mathrm{Precision}\left( C_{i} \right)= \frac{TP (C_{i})}{\mathrm{TP}\left( C_{i} \right)+FP (C_{i})}$ (1)

This is the precision for a single class, the total (macro) accuracy being as follows:

$\mathrm{Precision}\left( \mathrm{macro} \right)= \frac{1}{N} \sum_{i=1}^{N} Precision (C_{i})$ (2)

Recall (macro average):

$\mathrm{Recall}\left( C_{i} \right)= \frac{TP (C_{i})}{\mathrm{TP}\left( C_{i} \right)+FN (C_{i})}$ (3)

This is the recall for a single class, the total (macro) accuracy being as follows:

$\mathrm{Recall}\left( \mathrm{macro} \right)= \frac{1}{N} \sum_{i=1}^{N} Recall(C_{i})$ (4)

Accuracy:

$Accuracy= \frac{\sum_{i=1}^{N} \mathrm{TP}\left( C_{i} \right)+ TN\left( C_{i} \right)}{\sum_{i=1}^{N} \sum_{j=1}^{N} C_{I,j}}$ (5)

Being its definition all correct classifications/total number of data points.

F1-score:

$F1-score (macro)=\frac{2*Precision (macro)*Recall(macro)}{Precision (macro)+ Recall(macro)}$ (6)

considering a multi-class classification problem with a set containing the N different class labels Ci (I = 1, 2,...,N) where the variable j is the number of classes (thus, the denominator would be the whole number of data used in the sample set). Where TP, TN, FP and FN represent the number of true positives, true negatives, false positives and false negatives, respectively.

**Sample size estimation**

In general, machine learning algorithms require less data compared to deep learning models. A minimum of 1000 samples per category is considered adequate for machine learning algorithms. Our study covers three categories, with a distribution of 6428 breaths distributed into 2708 normal-mild (42.13%), 1535 moderate (23.88%) and 2185 severe (33.99%). To represent a broad spectrum of no-flow cases, breaths from 28 patients were selected.

To analyze whether the number of samples was sufficient, we used so-called progressive sampling (18), which uses the learning curve to determine the number of data needed for the model to learn. Thus, to assess the effectiveness of the applied models, we generated a learning curve that plotted sample size versus hit rate (see Figure E4). Thus, if at the last data points, the slope becomes flat or negative, it indicates that increasing the dataset is unlikely to significantly improve the hit rate (19). As shown in Figure E4, as the size of the training data set increases, the slope of the curve approaches zero. This phenomenon is especially noticeable when it is observed that a significant increase in the size of the training dataset results in a modest 3% improvement in accuracy, as seen in our evaluations.


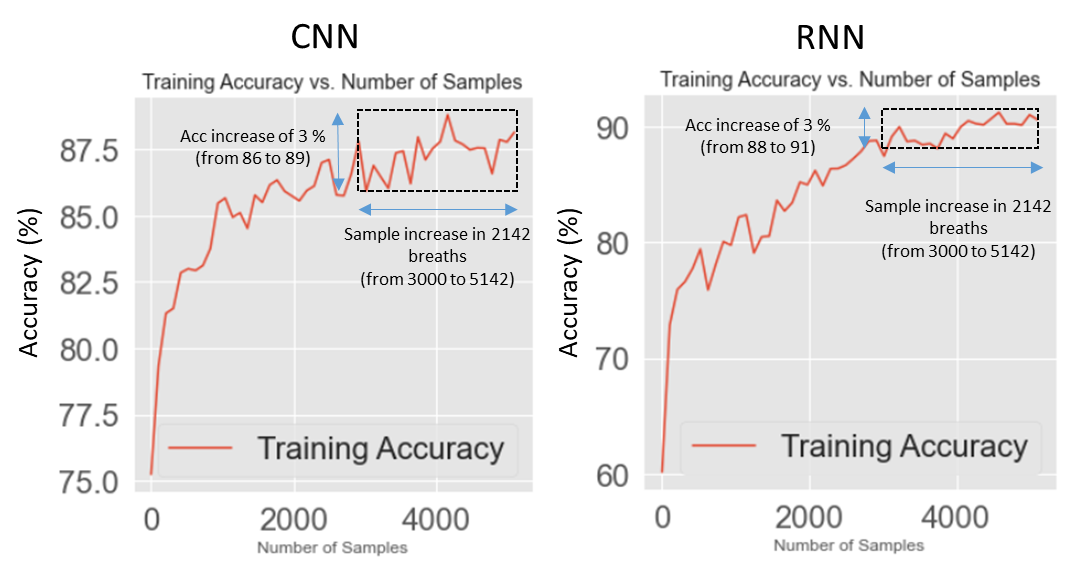


Figure E4. Learning curve of the sample size against the success rate. Models were trained with 80% (5142 samples) of dataset.

**Inter-expert agreement**

During the analysis phase of the annotation results we calculated the Fleiss’ kappa and it was moderate with k = 0.53 and standard error = 0.0028243.

This value indicates that the perfect agreement among the 5 experts is moderate. The figure below shows that in this scenario we should discard 58% of the breaths. In order to include as many as breaths as possible to train the algorithms, we explored the percentage of breaths with agreement as inter-expert agreement. The figure shows this analysis for 5/5, 4/5 and 3/5 annotators. Based on these results, we have decided to use the majority voting method (three of five expert’s agreement) to obtain the final labeling.

Figure E5. Inter-expert agreement calculated as the percentage of breaths with agreement in function of expert’s ratio (3/5, 4/5 and 5/5) with agreement. Perfect agreement among all experts (5/5) was found in 41.91% of breaths, while the agreement for 4/5 and 3/5 experts were found in 67.84% and 95.4% of breaths, respectively.

| **CNN** | | **1** | **2** | **3** | **4** | **5** | **6** | **7** | **8** | **9** | **10** | **11** | **12** | **13** | **14** | **15** |
| --- | --- | --- | --- | --- | --- | --- | --- | --- | --- | --- | --- | --- | --- | --- | --- | --- |
| **TRAIN** | *Precision* | 0,856 | 0,854 | 0,847 | 0,855 | 0,867 | 0,852 | 0,862 | 0,867 | 0,848 | 0,86 | 0,852 | 0,857 | 0,865 | 0,852 | 0,854 |
|  | *Recall* | 0,86 | 0,854 | 0,849 | 0,853 | 0,858 | 0,846 | 0,862 | 0,857 | 0,854 | 0,856 | 0,852 | 0,856 | 0,857 | 0,855 | 0,855 |
|  | *Accuracy* | 0,869 | 0,867 | 0,862 | 0,868 | 0,877 | 0,864 | 0,876 | 0,876 | 0,86 | 0,872 | 0,866 | 0,869 | 0,876 | 0,865 | 0,868 |
|  | *F1-score* | 0,858 | 0,854 | 0,848 | 0,854 | 0,862 | 0,849 | 0,862 | 0,861 | 0,85 | 0,858 | 0,854 | 0,856 | 0,861 | 0,853 | 0,854 |
| **TEST** | *Precision* | 0,872 | 0,868 | 0,863 | 0,869 | 0,876 | 0,863 | 0,877 | 0,875 | 0,866 | 0,872 | 0,866 | 0,871 | 0,874 | 0,867 | 0,87 |
|  | *Recall* | 0,869 | 0,867 | 0,862 | 0,868 | 0,877 | 0,864 | 0,876 | 0,876 | 0,86 | 0,872 | 0,866 | 0,869 | 0,876 | 0,865 | 0,868 |
|  | *Accuracy* | 0,869 | 0,867 | 0,862 | 0,868 | 0,877 | 0,864 | 0,876 | 0,876 | 0,86 | 0,872 | 0,866 | 0,869 | 0,876 | 0,865 | 0,868 |
|  | *F1-score* | 0,87 | 0,867 | 0,862 | 0,868 | 0,876 | 0,864 | 0,876 | 0,876 | 0,862 | 0,872 | 0,866 | 0,87 | 0,874 | 0,866 | 0,869 |
| **RNN** | | **1** | **2** | **3** | **4** | **5** | **6** | **7** | **8** | **9** | **10** | **11** | **12** | **13** | **14** | **15** |
| **TRAIN** | *Precision* | 0,872 | 0,869 | 0,865 | 0,862 | 0,864 | 0,87 | 0,861 | 0,866 | 0,868 | 0,87 | 0,861 | 0,864 | 0,86 | 0,864 | 0,87 |
|  | *Recall* | 0,864 | 0,867 | 0,868 | 0,851 | 0,855 | 0,868 | 0,859 | 0,858 | 0,856 | 0,87 | 0,854 | 0,859 | 0,86 | 0,852 | 0,861 |
|  | *Accuracy* | 0,883 | 0,883 | 0,879 | 0,874 | 0,876 | 0,883 | 0,876 | 0,88 | 0,88 | 0,883 | 0,876 | 0,878 | 0,873 | 0,876 | 0,882 |
|  | *F1-score* | 0,867 | 0,868 | 0,866 | 0,855 | 0,858 | 0,869 | 0,86 | 0,861 | 0,86 | 0,87 | 0,856 | 0,861 | 0,86 | 0,856 | 0,865 |
| **TEST** | *Precision* | 0,882 | 0,882 | 0,881 | 0,872 | 0,874 | 0,884 | 0,875 | 0,878 | 0,877 | 0,884 | 0,873 | 0,876 | 0,875 | 0,873 | 0,88 |
|  | *Recall* | 0,883 | 0,883 | 0,879 | 0,874 | 0,876 | 0,883 | 0,876 | 0,88 | 0,88 | 0,883 | 0,876 | 0,878 | 0,873 | 0,876 | 0,882 |
|  | *Accuracy* | 0,883 | 0,883 | 0,879 | 0,874 | 0,876 | 0,883 | 0,876 | 0,88 | 0,88 | 0,883 | 0,876 | 0,878 | 0,873 | 0,876 | 0,882 |
|  | *F1-score* | 0,882 | 0,883 | 0,88 | 0,872 | 0,874 | 0,883 | 0,875 | 0,878 | 0,877 | 0,883 | 0,874 | 0,877 | 0,874 | 0,873 | 0,88 |

Table E1. Performance metrics (precision, recall, accuracy and F1-score) obtained of implemented models: convolutional neural networks (CNN) and recurrent neural networks (RNN). The metrics are shown for noth training and test procedure. The training and test procedure was repeated 15 times to attain a less biased estimate of model performance.

**Figure E6**. Distribution of (a) inspiratory time (T_i_), (b) peak airflow, (c) expiratory time, (d) respiratory rate, (e) tidal volume and (f) PEEP for each inspiratory effort pattern. The median value of the breath developed for each patient is shown as a small circle. The bigger circles show the average number across all patients. The ventilator inspiratory time (T_i_) was automatically determined by zero crossing the airflow tracing. There are no differences between breath categories


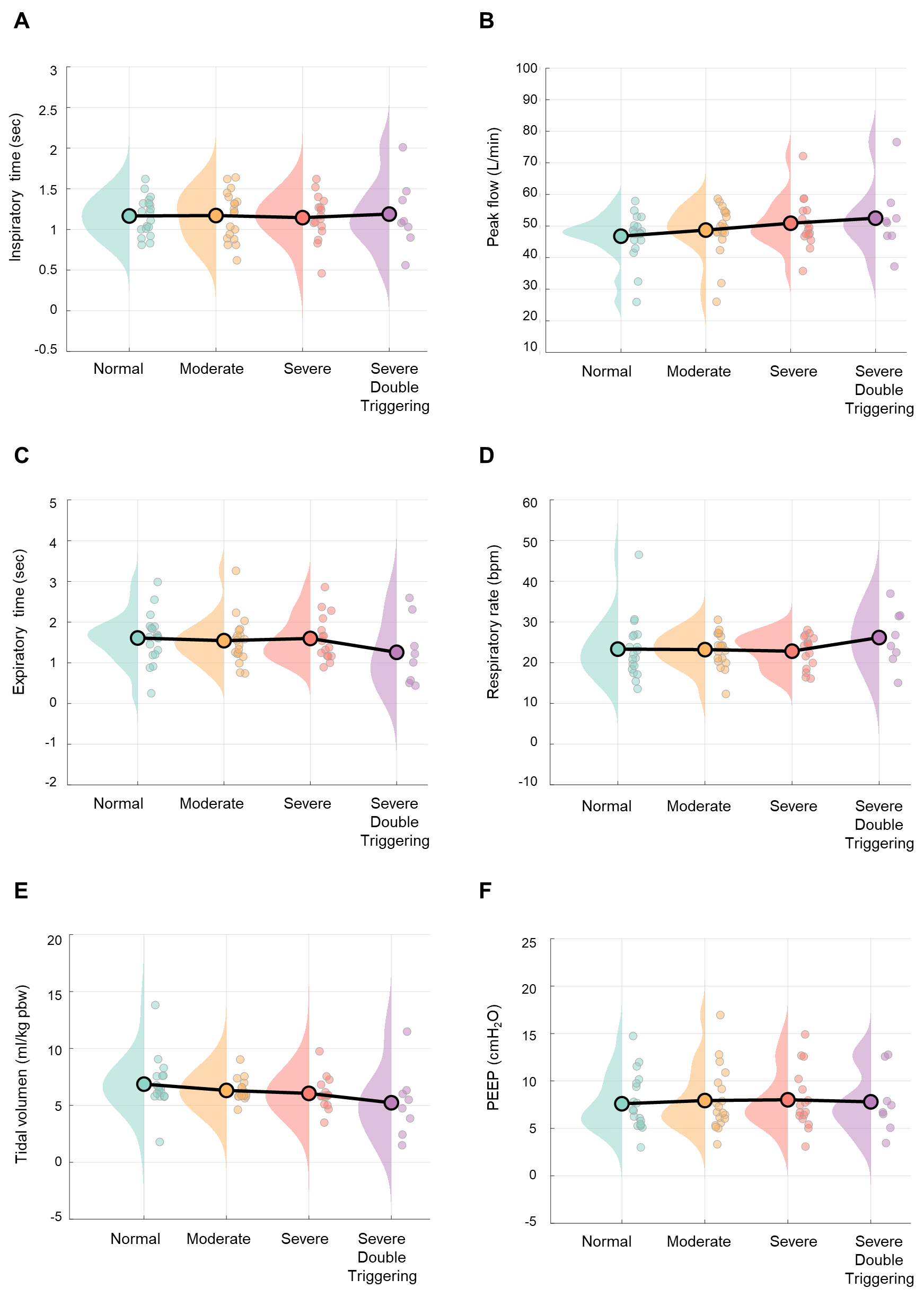


Table E2. Distribution of inspiratory time, peak airflow, expiratory time, respiratory rate, tidal volume and PEEP for each inspiratory effort pattern. Data are expressed as median and IQR across subjects of the within-subject medians

|  | **Normal** | **Moderate** | **Severe** | **Severe with Double-Triggering** |
| --- | --- | --- | --- | --- |
| **Inspiratory time** | 1.2 (1-1.3) | 1.2 (0.9-1.4) | 1.2 (1-1.3) | 1.1 (1-1.4) |
| **Peak airflow** | 48 (45.5-50.1) | 49.6 (47.8-54.3) | 49.3 (47-54.9) | 51.3 (46.9-54.9) |
| **Expiratory time** | 1.6 (1.2-1.9) | 1.6 (1.2-1.8) | 1.5 (1.2-2) | 1.1 (0.5-1.9) |
| **Respiratory Rate** | 22.1 (18.6-26.7) | 24.1 (20.5-27) | 24.3 (19.2-26.2) | 25.4 (21.7-31.5) |
| **Tidal volume** | 6.4 (5.8-7.6) | 6 (5.8-6.6) | 5.9 (5.3-6.7) | 5.1 (3.1-6.2) |
| **PEEP** | 6.9 (5.4-9.7) | 6.7 (5.7-9.7) | 7.1 (6.2-9.7) | 7.1 (5.8-10.2) |

**Secondary analysis of BEARDS patients with esophageal pressure measurements**

This secondary analysis involves assessing how changes in airway pressure (P_aw_) deformation relate to the level of inspiratory effort as evaluated by changes in esophageal pressure (ΔP_es_). The main goal is to understand how alterations in P_aw_, potentially resulting from variations in mechanical ventilation settings or patient-specific factors, correspond to changes in inspiratory effort. A total of 480 breaths from 8 patients were considered.

Table E3. Relationship between different levels of P_aw_ deformation and the level of inspiratory effort evaluated with the ΔP_es_.

| ΔP_es_ **(cmH_2_O)** | **NORMAL**  **(number of breaths)**  **n=42** | **NORMAL (%)** | **MODERATE**  **(number of breaths)**  **n=229** | **MODERATE (%)** | **SEVERE**  **(number of breaths)**  **n=250** | **SEVERE (%)** |
| --- | --- | --- | --- | --- | --- | --- |
| >8 | 1 | 2,40 | 81 | 35,40 | 237 | 94,80 |
| >10 | 0 | 0 | 11 | 4,80 | 186 | 74,40 |
| >15 | 0 | 0 | 0 | 0 | 93 | 37,20 |

*Paw = airway pressure; Pes = esophageal pressure; % is referenced to the total number of breaths (n = 480 breaths)*

**References**

1. Nath A, Subbiah K. The role of pertinently diversified and balanced training as well as testing data sets in achieving the true performance of classifiers in predicting the antifreeze proteins. Neurocomputing. 2018 Jan 10;272:294–305.

2. Wei Q, Dunbrack RL. The Role of Balanced Training and Testing Data Sets for Binary Classifiers in Bioinformatics. PLoS One. 2013;8(7):67863.

3. Smirnov D, Nguifo EM. Time Series Classification with Recurrent Neural Networks. ECML/PKDD Work Adv Anal Learn Temporal Data. 2018;1–8.

4. Hüsken M, Stagge P. Recurrent neural networks for time series classification. Neurocomputing. 2003;50:223–35.

5. Squartini S, Hussain A, Piazza F. Preprocessing based solution for the vanishing gradient problem in recurrent neural networks. In: Proceedings of the 2003 International Symposium on Circuits and Systems, 2003 ISCAS ’03. IEEE; 2003. p. V-713-V–716.

6. Staudemeyer RC, Morris ER. Understanding LSTM -- a tutorial into Long Short-Term Memory Recurrent Neural Networks. Neural Evol Comput. 2019 Sep 12;1–42.

7. Reddy BK, Delen D. Predicting hospital readmission for lupus patients: An RNN-LSTM-based deep-learning methodology. Comput Biol Med. 2018 Oct 1;101:199–209.

8. Barbieri S, Kemp J, Perez-Concha O, Kotwal S, Gallagher M, Ritchie A, et al. Benchmarking Deep Learning Architectures for Predicting Readmission to the ICU and Describing Patients-at-Risk. Sci Rep. 2020 Jan 24;10(1):1111.

9. Ashfaq A, Sant’Anna A, Lingman M, Nowaczyk S. Readmission prediction using deep learning on electronic health records. J Biomed Inform. 2019;97(October 2018):103256.

10. Saxena A. An Introduction to Convolutional Neural Networks. Int J Res Appl Sci Eng Technol. 2022 Dec 31;10(12):943–7.

11. Li Z, Liu F, Yang W, Peng S, Zhou J. A Survey of Convolutional Neural Networks: Analysis, Applications, and Prospects. IEEE Trans Neural Networks Learn Syst. 2022 Dec 1;33(12):6999–7019.

12. Khalilpour S, Ranjbar A, Menhaj MB, Sandooghdar A. Application of 1-D CNN to Predict Epileptic Seizures using EEG Records. In: 2020 6^th^ International Conference on Web Research (ICWR). IEEE; 2020. p. 314–8.

13. Oh SL, Ng EYK, Tan RS, Acharya UR. Automated diagnosis of arrhythmia using combination of CNN and LSTM techniques with variable length heart beats. Comput Biol Med. 2018 Nov 1;102:278–87.

14. Prifti E, Fall A, Davogustto G, Pulini A, Denjoy I, Funck-Brentano C, et al. Deep learning analysis of electrocardiogram for risk prediction of drug-induced arrhythmias and diagnosis of long QT syndrome. Eur Heart J. 2021 Oct 7;42(38):3948–61.

15. Gu J, Wang Z, Kuen J, Ma L, Shahroudy A, Shuai B, et al. Recent advances in convolutional neural networks. Pattern Recognit. 2018 May 1;77:354–77.

16. Zhao B, Lu H, Chen S, Liu J, Wu D. Convolutional neural networks for time series classification. J Syst Eng Electron. 2017 Feb 20;28(1):162–9.

17. Ismail Fawaz H, Forestier G, Weber J, Idoumghar L, Muller P-A. Deep learning for time series classification: a review. Data Min Knowl Discov. 2019;33(4):917–63.

18. Mukherjee S, Tamayo P, Rogers S, Rifkin R, Engle A, Campbell C, Golub TR, Mesirov JP. Estimating dataset size requirements for classifying DNA microarray data. J Comput Biol. 2003;10(2):119-42. doi: 10.1089/106652703321825928. PMID: 12804087.

19. T. Viering and M. Loog, "The Shape of Learning Curves: A Review" in IEEE Transactions on Pattern Analysis & Machine Intelligence, vol. 45, no. 06, pp. 7799-7819, 2023.

20. Markoulidakis I, Rallis I, Georgoulas I, Kopsiaftis G, Doulamis A, Doulamis N. Multiclass Confusion Matrix Reduction Method and Its Application on Net Promoter Score Classification Problem. Technologies. 2021; 9(4):81.
